# Thoracic duct outflow obstruction increases thoracic duct pressure above central venous pressure supporting thoracic duct decompression

**DOI:** 10.1016/j.jvssci.2026.100425

**Published:** 2026-05-08

**Authors:** Takuji Maruyama, Shuji Kariya, Miyuki Nakatani, Yutaka Ueno, Yasuyuki Ono, Yuki Tanaka, Kanji Sugiura, Noboru Tanigawa

**Affiliations:** Department of Radiology, Kansai Medical University, Osaka, Japan

**Keywords:** Embolization, Lymphangiography, Lymphatic pressure, Swine model, Thoracic duct

## Abstract

**Objective:**

Thoracic duct outflow obstruction is a recognized cause of refractory lymphatic leakage; however, the physiological consequences of such obstruction on thoracic duct pressure and its relationship to central venous pressure have not been fully characterized in vivo. This study aimed to evaluate changes in thoracic duct pressure and the pressure gradient between the thoracic duct and the central venous system in an experimental model of thoracic duct outflow obstruction.

**Methods:**

In a swine model, thoracic duct pressure and central venous pressure were directly measured using intravascular catheters positioned at matched vertebral levels. Thoracic duct embolization was performed at the upper thoracic level using coils and cyanoacrylate and was used as an experimental model of thoracic duct outflow obstruction. Pressure measurements were obtained before embolization and after confirmation of complete thoracic duct occlusion. Changes in thoracic duct pressure, central venous pressure, and the pressure relationship between the two systems were analyzed using paired statistical comparisons.

**Results:**

Thoracic duct outflow obstruction resulted in a significant increase in upstream thoracic duct pressure, with a median increase of approximately 6 to 8 mmHg compared with preobstruction values. Following obstruction, thoracic duct pressure consistently exceeded central venous pressure, creating a pressure gradient indicative of lymphatic hypertension. In contrast, central venous pressure did not change significantly after thoracic duct obstruction. No consistent cranio-caudal longitudinal pressure gradient along the thoracic duct was identified.

**Conclusions:**

Thoracic duct outflow obstruction induces lymphatic hypertension, as demonstrated by an increase in thoracic duct pressure relative to central venous pressure. This experimentally demonstrated pressure gradient provides physiological support for decompressive interventions by demonstrating a pressure gradient favorable for lymphatic drainage into the venous system. By directly quantifying intralymphatic and venous pressures in vivo, this study offers mechanistic insight into the pathophysiology of lymphatic leakage associated with thoracic duct obstruction and may help inform treatment selection between occlusive and decompressive strategies in lymphatic interventions.

**Clinical Relevance:**

Thoracic duct outflow obstruction is a cause of lymphatic leakage, yet the consequences of obstruction on thoracic duct pressure and its relationship to central venous pressure have remained unclear. In this experimental study, in vivo measurements demonstrated that thoracic duct outflow obstruction results in an increase in thoracic duct pressure, creating a pressure gradient relative to central venous pressure. This pressure relationship reflects lymphatic hypertension caused by outflow obstruction and supports decompressive interventions aimed at restoring lymphatic outflow.

**Article Highlights**

• **Type of Research:** In vivo experimental animal study
• **Key Findings:** Thoracic duct outflow obstruction was experimentally created by thoracic duct embolization in a swine model. Obstruction increased upstream thoracic duct pressure by a median of 6 to 8 mmHg and created a pressure environment indicative of lymphatic hypertension, whereas central venous pressure remained unchanged. These findings support the physiological rationale for decompressive interventions by establishing a pressure relationship favorable for lymphatic drainage into the venous system.
• **Take Home Message:** Thoracic duct outflow obstruction induces lymphatic hypertension, providing a physiological basis for thoracic duct decompression in selected patients with lymphatic leakage.

The lymphatic system drains interstitial fluid to help maintain tissue fluid homeostasis, transports immune cells, and conveys dietary lipids from the intestine.^1^ If the lymphatic system is damaged or obstructed, lymphatic fluid may leak from the lymphatic vasculature.^2^, ^3^, ^4^ High-output leakage can lead to immunodeficiency and malnutrition and may be life-threatening.^1^, ^2^, ^3^ Thresholds commonly used when considering definitive intervention—surgical or interventional radiology—include ≥500 mL/day in the cervical or abdominal regions^4^^,^^5^ and ≥1000 mL/day in the thoracic cavity.^2^ Definitive interventions for lymphatic leakage include thoracic duct ligation and thoracic duct embolization; however, the physiological consequences of thoracic duct outflow obstruction, particularly changes in intraductal pressure, have not been fully elucidated.

Clinically, obstruction of the thoracic duct is known to be one of the causes of lymphatic leakage. For example, ligation of the thoracic duct during esophagectomy has been reported to contribute to the development of chylothorax.^6^^,^^7^ In addition, thoracic duct obstruction has also been reported as a potential cause in conditions characterized by lymphatic leakage, including plastic bronchitis,^8^ chylous ascites,^9^ protein-losing enteropathy,^10^ and chyluria.^11^ Physiologically, obstruction of the thoracic duct may increase pressures in the duct and in connected lymphatic channels upstream (caudal denotes the upstream direction) of the obstruction. If these pressures exceed the upper limit of the pressure tolerance of the thoracic duct, lymphatic vessels, or collateral pathways, wall disruption or exudative extravasation is thought to occur, resulting in lymphatic leakage.^2^^,^^12^^,^^13^

Decompressive procedures that lower thoracic duct and lymphatic pressures may be effective in treating lymphatic leakage attributable to thoracic duct obstruction. The thoracic duct normally drains into the venous system via the venous angle, maintaining pressure balance. Accordingly, reconstruction of the native venous outflow or creation of a new venous outflow from the thoracic duct may achieve decompression. Procedures reported for this purpose include thoracic–duct plasty, which recanalizes the obstructed duct and restores drainage to the venous angle,^9^^,^^14^ and thoracic duct–venous bypass, which creates an anastomosis between the thoracic duct and a systemic vein.^13^^,^^15^

To support the mechanistic basis of lymphatic leakage in thoracic duct obstruction and the physiological rationale for decompressive procedures, it is necessary to confirm in vivo whether obstruction leads to sustained elevation of thoracic duct pressure and alters the pressure relationship between the thoracic duct and the central venous system.

Accordingly, the purpose of this study was to evaluate changes in thoracic duct pressure upstream of the embolization site during thoracic duct embolization and to examine the pressure gradient between thoracic duct and central venous pressures before and after embolization.

## Methods

In this study, the caudal direction with respect to lymph flow is referred to as “upstream,” and the cranial direction as “downstream.” Thoracic duct embolization was used in this study as an experimental model of thoracic duct outflow obstruction.

### Experimental procedure

**Fig 1:**
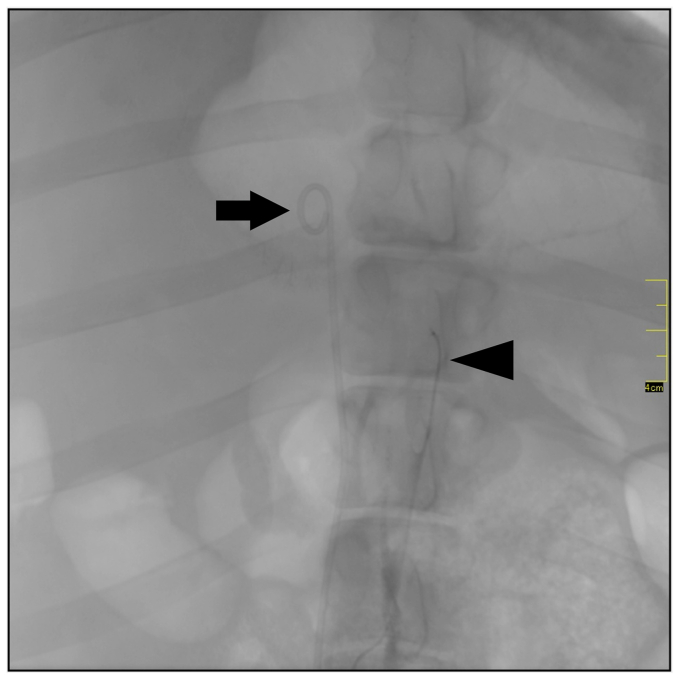
Fluoroscopic image during pressure measurements. A pigtail catheter is positioned in the central venous system and a microcatheter in the thoracic duct. Thoracic duct pressure and central venous pressure were recorded with the catheter openings aligned at the same vertebral level. *Black arrow*, pigtail catheter in central vein; *black arrowhead*, microcatheter in thoracic duct.

**Supplementary Fig 1:**
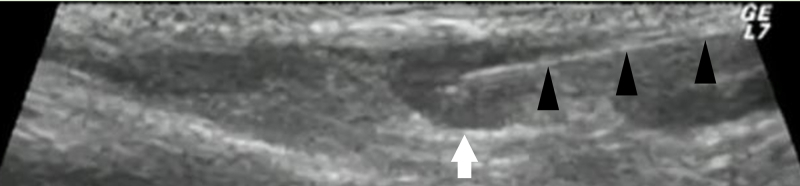


**Supplementary Fig 2:**
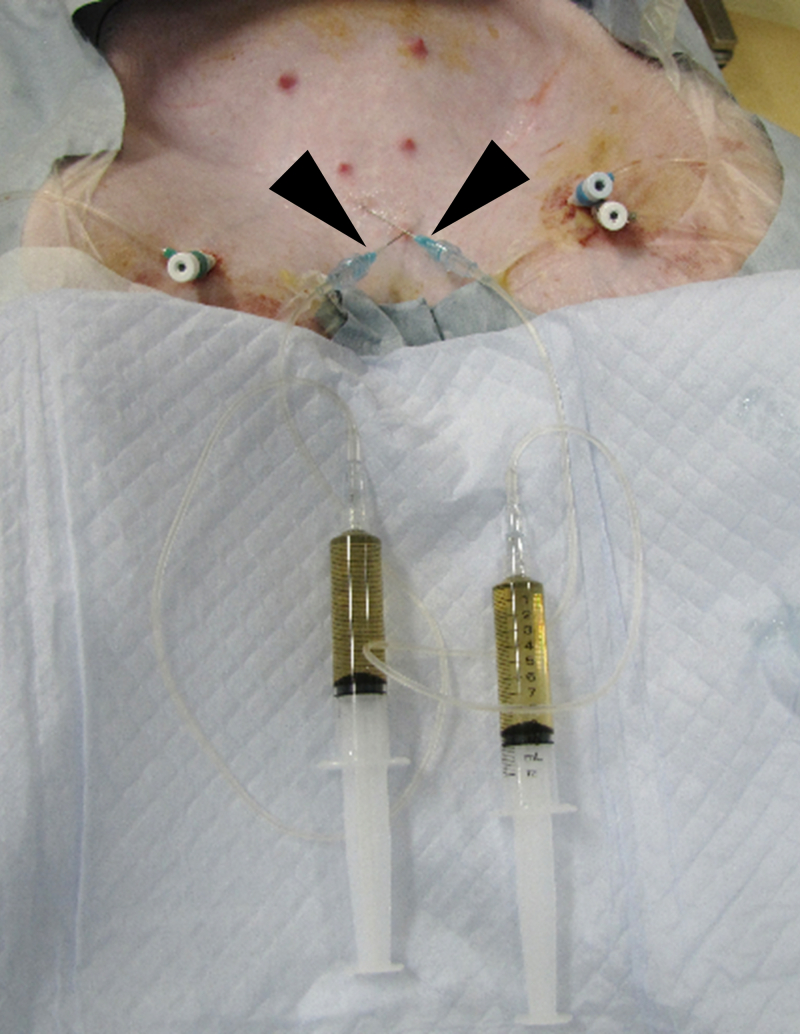


**Supplementary Fig 3:**
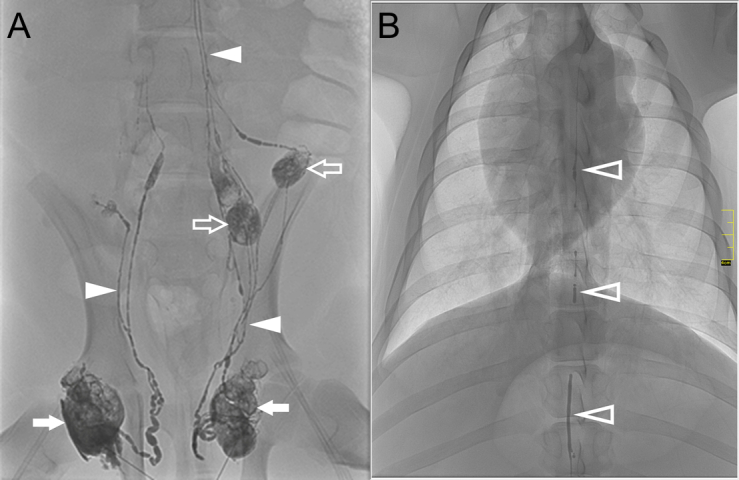


All procedures were performed in an American Association for Accreditation of Laboratory Animal Care International-accredited facility and were approved by the institutional Animal Care and Use Committee (approval nos. S20–016 and S24–015). All procedures complied with the Guide for the Care and Use of Laboratory Animals (National Research Council) and the Animal Research: Reporting of In Vivo Experiments guidelines. Ten female swine (12-15 weeks of age; 39-45 kg) were studied. Swine were positioned supine, anesthesia was induced with isoflurane, the trachea was intubated, and mechanical ventilation was maintained using pressure-controlled ventilation (mean pressure of airway, 17-26 cmH_2_O; respiratory rate, 15-22 breaths/min; positive end-expiratory pressure, 5 cm H_2_O). Although some ventilator settings were not consistently recorded, all swine were managed under similar ventilatory conditions. Vital signs were monitored continuously throughout anesthesia. Imaging was performed with an ultrasound system (Logiq 7, GE Medical Systems) equipped with a 12-MHz linear array transducer (M12 L GII, GE Medical Systems) and with a flat-panel angiography system (Azurion 7 C20, Royal Philips). Introducer sheaths (5F or 6F, 11 cm; Super Sheath, Medikit) were placed in the right external jugular vein and in the right and left femoral veins. An additional arterial sheath (5F or 8F, 11 cm; Super Sheath, Medikit) was placed via the right or left femoral artery. Two sets were prepared, each consisting of a 10-mL syringe connected to diethylhexyl phthalate-free tubing, which in turn was connected at the opposite end to a 23-gauge, 7-cm Cathelin needle (all Terumo Corporation). Both sets were filled with ethiodized oil (Lipiodol; ethyl esters of iodinated poppy-seed oil fatty acids; Guerbet Japan). Both superficial inguinal lymph nodes were accessed under ultrasound guidance using the Cathelin needle, and ethiodized oil was injected into each node as a contrast agent (Supplementary Figs 1 and 2, online only). The contrast agent traversed the lymphatic vessels in an antegrade fashion and opacified the thoracic duct (Supplementary Fig 3, A and B, online only). The thoracic duct was then punctured percutaneously via an anterior transabdominal approach under fluoroscopic guidance at a level caudal to the 12th thoracic vertebra, using a 22-gauge, 20-cm Chiba needle (Angiotech). A guidewire (0.014 inch, 200 cm, Synchro-14, Boston Scientific Japan; or 0.018 inch, 300 cm, V-18 Control, Boston Scientific Japan) was advanced into the thoracic duct, followed by a 1.7F, 130-cm microcatheter (Breakthrough, Boston Scientific Japan) introduced over the wire. A 5F, 110-cm pigtail catheter (Medikit) was advanced through the right femoral venous sheath, and the tip was positioned in the central venous system. Patency of the thoracic duct and the central venous system at the measurement level and downstream was confirmed under fluoroscopy; no angiographic evidence of obstruction or impaired transit was identified. Iodinated contrast medium (iopamidol 300, 300 mg I/mL; Fuji Pharma Co, Ltd) was used for angiography and thoracic ductography. Thoracic duct pressure was measured with the microcatheter, and central venous pressure was measured with the pigtail catheter (Fig 1). Pressures were monitored using a polygraph system (FCL-1000; Fukuda Denshi) equipped with a pressure transducer (DTX Plus; Merit Medical). The transducer was zeroed at the level of the right atrium. Under mechanical ventilation, the representative value was recorded once the readings had remained stable for approximately five consecutive cardiac cycles. Thoracic vertebral levels were identified based on fluoroscopic findings, and thoracic duct and central venous pressures were measured at thoracic vertebral levels T5 to T13. For swine 1 through 4, because the procedure was still in its initial phase, pressure was measured only at the T12 level. For swine 5 through 10, as the procedure became more familiar, the tip positions of the thoracic duct catheter and the central venous catheter were adjusted to obtain pressure measurements at multiple levels from T5 to T13. In swine 9, the thoracic duct coursed on the left side, making percutaneous puncture upstream of T12 difficult; therefore, the duct was punctured at T12. Catheter manipulation was also challenging in this swine, and measurements of thoracic duct and central venous pressures were limited to the T9 and T10 levels.

**Fig 2:**
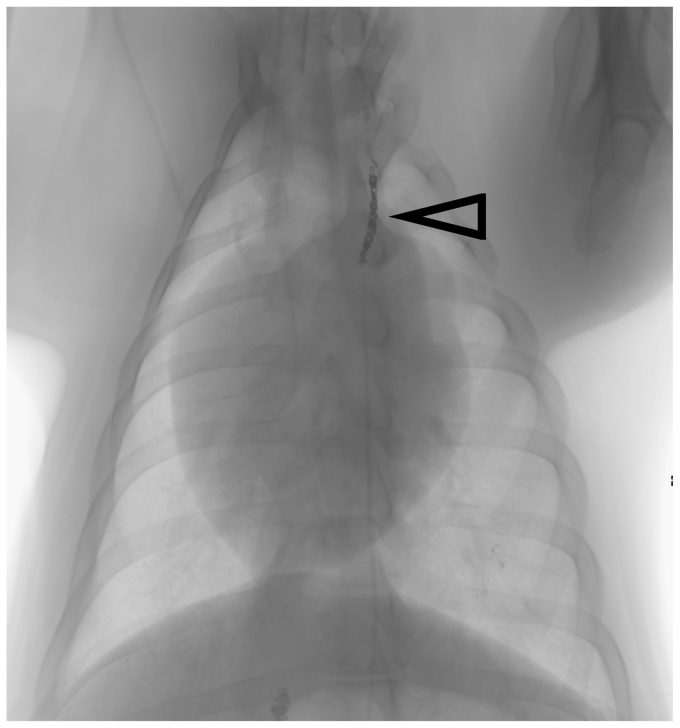
Fluoroscopic image after thoracic duct embolization at upper thoracic vertebral levels. Embolic material within the thoracic duct is shown. *Black hollow arrowhead*, detachable coils and a cyanoacrylate cast within the thoracic duct.

Transcatheter embolization of the thoracic duct was performed downstream to the T4 level (Fig 2). Embolic materials included detachable coils (Interlock-18 and IDC in complex helical and diamond configurations; Boston Scientific Japan) and 2-octyl cyanoacrylate adhesive (Dermabond Advanced or Dermabond Mini; Johnson & Johnson K.K.). After ductal occlusion was confirmed by lymphangiography, thoracic duct and central venous pressures were measured upstream to the embolized segment using the same protocol as before embolization. At the end of the experiment, euthanasia was performed by continuous inhalation of an isoflurane overdose via an endotracheal tube under deep anesthesia until cardiac arrest was confirmed, in accordance with the 2013 American Veterinary Medical Association Guidelines for the Euthanasia of Animals and an Institutional Animal Care and Use Committee–approved protocol. All procedures were performed to minimize pain and distress.

### Statistical analysis

We evaluated the change in thoracic duct pressure before vs after embolization, the change in central venous pressure before vs after embolization, and the difference between thoracic duct and central venous pressures. For each swine, the average of all thoracic duct pressure measurements and the average of all central venous pressure measurements were calculated separately across the thoracic vertebral levels at which measurements were calculated, which were used for analysis. In addition, pressures at the T12 level were also analyzed in parallel as the representative single-vertebral level, because this level had valid measurements in seven of 10 swine, the largest number among the vertebral levels. The Wilcoxon signed-rank test was used as the primary analysis because the normality of the distribution of the paired differences could not be reliably confirmed. In addition, the mean of the paired differences and corresponding 95% confidence intervals (CIs) were calculated based on paired *t* methods to quantify the effect size. Predefined exclusion criteria were persistent hemodynamic instability during the procedure and abnormally elevated central venous pressure following embolization, both of which could render pressure measurements unreliable. We additionally examined whether a longitudinal cranio-caudal pressure gradient was present within the thoracic duct. Measurements of thoracic duct pressure at vertebral levels T5 to T13 were included in the analysis. For each swine, thoracic vertebral level was regressed against thoracic duct pressure, and the regression slope (mmHg per vertebral level) was defined as the intraductal pressure gradient; positive values indicate higher pressures upstream, and negative values indicate higher pressures downstream. Swine for which measurements were obtained at two or fewer vertebral levels were excluded because linear regression analysis could not be performed. The Wilcoxon signed-rank test was used to assess whether the median slope differed from zero in the pre- and post-embolization conditions. All tests were two-tailed with a significance level of .05. Analyses were performed using JMP Pro 18 (SAS Institute Japan Ltd). Because this was a within-animal before-and-after comparison, randomization was not applicable. In addition, no formal sample size calculation was performed because this was an exploratory physiological study.

## Results

A total of 10 swine underwent the procedure. Two swine were excluded from analysis: one because of sustained hypotension and bradycardia with persistent hemodynamic instability during the procedure, and the other because of abnormally elevated central venous pressure after embolization, interpreted as right-heart strain. Data from the remaining eight swine were analyzed.

**Fig 3:**
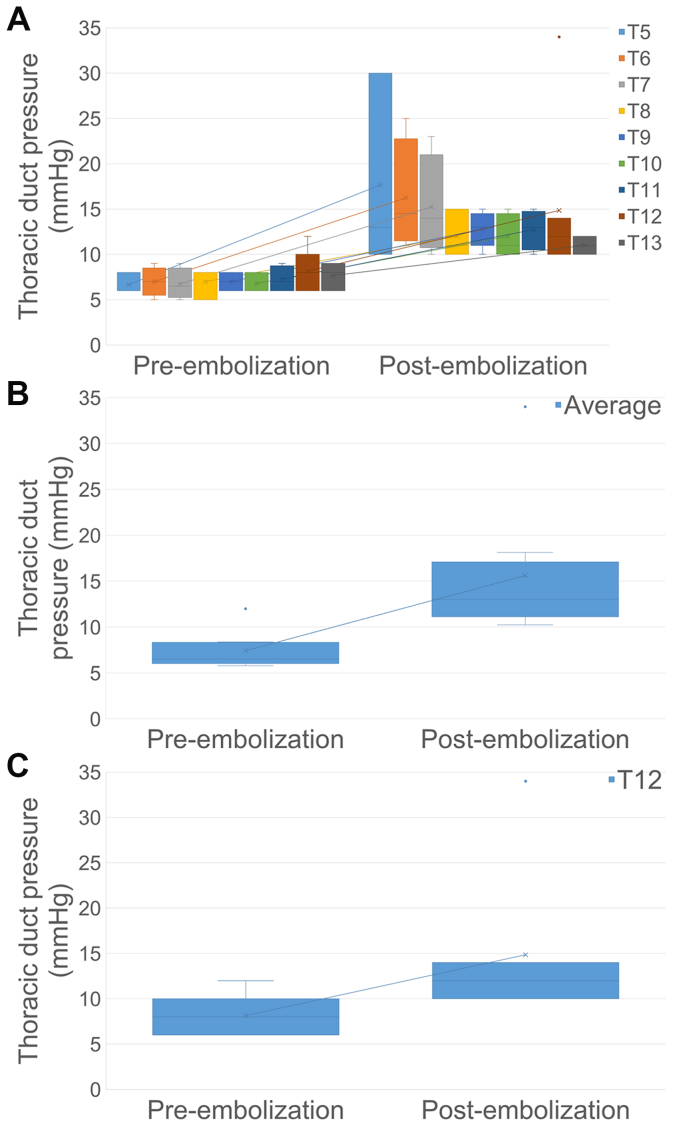
Thoracic duct pressures before and after thoracic duct embolization. **(A)** Thoracic duct pressure at each vertebral level from T5 to T13, compared before and after embolization. **(B)** The average thoracic duct pressure across vertebral levels T5 to T13 in each swine. Thoracic duct pressure increased after embolization (*P* = .0078). **(C)** Thoracic duct pressure at vertebral level T12. Thoracic duct pressure increased after embolization (*P* = .0313).

Individual pressure measurements for each swine are provided in the Supplementary Table (online only). Summary statistics are presented in the Table. Thoracic duct pressure increased after embolization across vertebral levels (Fig 3, *A*). The median of the averages of thoracic duct pressure measurements for each swine increased from 6.39 mmHg (range, 5.8-12 mmHg) to 12.94 mmHg (range, 10.2-34 mmHg) after embolization (*P* = .0078) (Fig 3, *B*). The mean increase in thoracic duct pressure was 8.2 mmHg (95% CI, 3.1-13.3 mmHg). At the T12 level, thoracic duct pressures likewise increased from 8 mmHg (range, 6-12 mmHg) to 12 mmHg (range, 10-34 mmHg) (*P* = .0313) (Fig 3, *C*). The mean increase in thoracic duct pressure was 6.71 mmHg (95% CI, 0.09-13.33 mmHg).

**Table:** Summary of thoracic duct and central venous pressures before and after thoracic duct embolization

	Pre-embolization	Postembolization	*P* value
Thoracic duct pressure, mmHg			
Median (range)	6.39 (5.8-12)	12.94 (10.2-34)	.0078
Mean change (95% CI)	–	+8.2 (3.1-13.3)	
Central venous pressure, mmHg			
Median (range)	4.65 (2-10)	4.86 (2-8)	.8438
Mean change (95% CI)	–	−0.15 (−1.13 to 0.84)	

*CI*, Confidence interval.

Central venous pressure did not change significantly after embolization. The median of the averages of central venous pressure measurements was 4.65 mmHg (range, 2-10 mmHg) pre-embolization and 4.86 mmHg (range, 2-8 mmHg) postembolization (*P* = .8438). The mean increase in central venous pressure was −0.15 mmHg (95% CI, −1.13 to 0.84 mmHg). At T12, the median central venous pressure was 4 mmHg both pre-embolization (range, 2-7 mmHg) and postembolization (range, 2-6 mmHg) (*P* = 1.0000). The mean increase in central venous pressure was −0.15 mmHg (95% CI, −0.97 to 0.69 mmHg).

**Fig 4:**
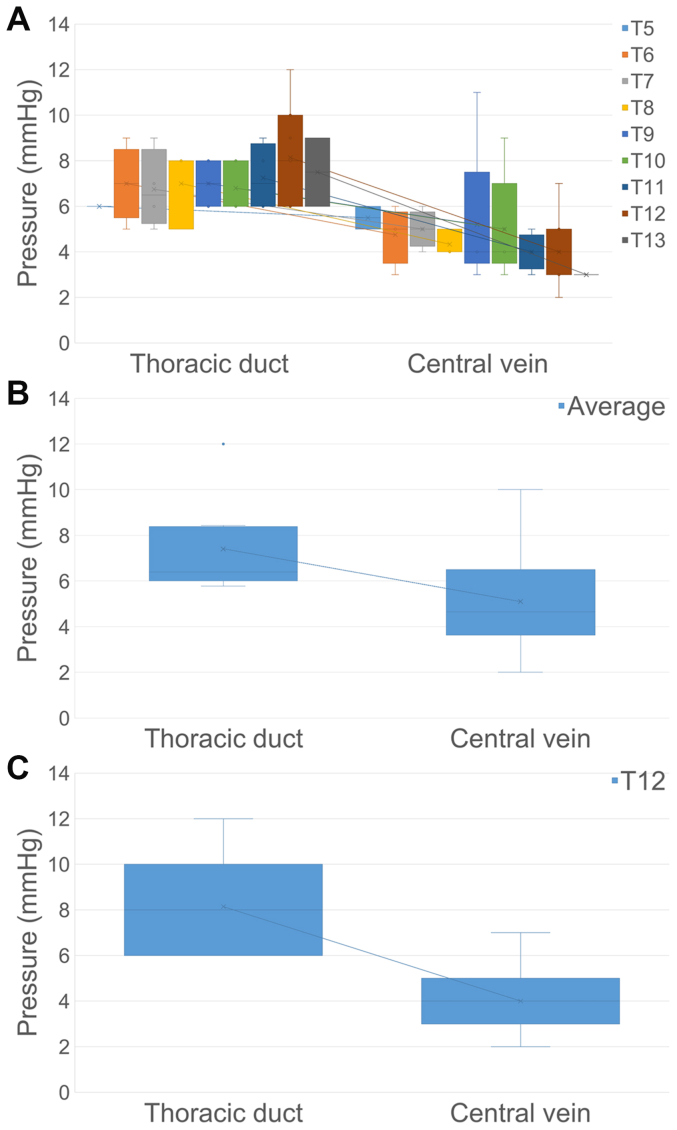
Pre-embolization comparisons of thoracic duct and central venous pressures. **(A)** Thoracic duct pressure and central venous pressure at each vertebral level from T5 to T13. **(B)** Comparison of average thoracic duct pressure and central venous pressure across vertebral levels T5 to T13. No significant difference was observed (*P* = .0781). **(C)** Comparison at vertebral level T12. Thoracic duct pressure was higher than central venous pressure (*P* = .0313).

**Fig 5:**
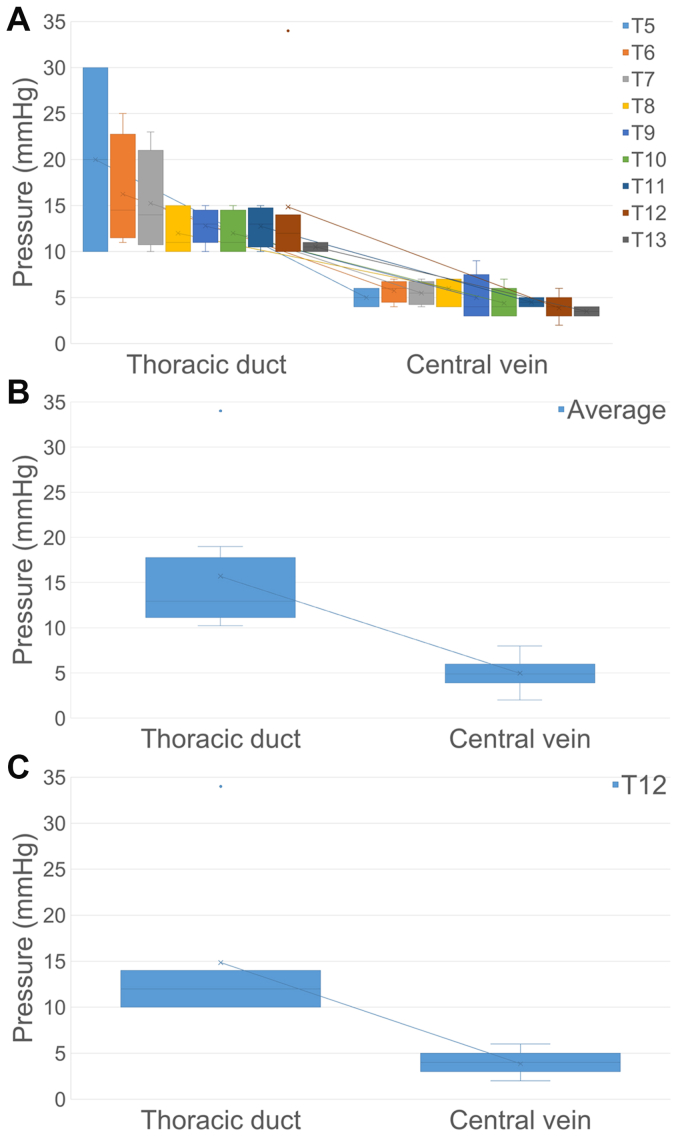
Postembolization comparisons of thoracic duct and central venous pressures. **(A)** Thoracic duct pressure and central venous pressure at each vertebral level from T5 to T13. **(B)** Comparison of average thoracic duct pressure and central venous pressure across vertebral levels T5 to T13. Thoracic duct pressure was significantly higher than central venous pressure (*P* = .0078). **(C)** Comparison at vertebral level T12. Thoracic duct pressure was higher than central venous pressure (*P* = .0156).

Vertebral level pressure profiles before embolization are shown in Fig 4, *A*. There was no significant difference between the average thoracic duct pressure and the average central venous pressure (*P* = .0781) (Fig 4, *B*), whereas at T12, the thoracic duct pressure was higher (*P* = .0313) (Fig 4, *C*). Vertebral level pressure profiles after embolization are shown in Fig 5, *A*. Thoracic duct pressure exceeded central venous pressure on both the average (*P* = .0078) (Fig 5, *B*) and at T12 (*P* = .0156) (Fig 5, *C*).

**Fig 6:**
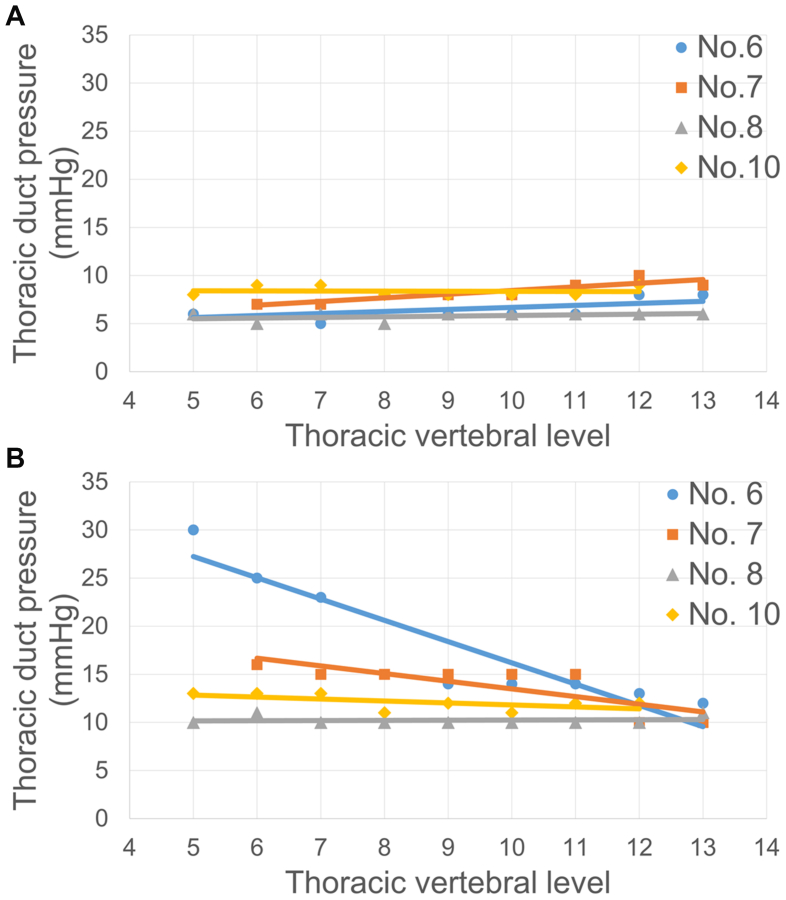
Thoracic duct pressure by vertebral level before and after embolization in swine with ≥3 measured levels. **(A)** Before embolization: thoracic duct pressure at vertebral levels T5 to T13 in four swine (Nos. 6, 7, 8, and 10). The median slope did not differ from 0 (+0.08 mmHg per vertebral level; *P* = .25). **(B)** After embolization: thoracic duct pressure at vertebral levels T5 to T13 in the same four swine. The median slope also did not differ from 0 (−0.20 mmHg per vertebral level; *P* = .09).

A longitudinal cranio-caudal pressure gradient within the thoracic duct was assessed in four swine. Swine with measurements at two or fewer vertebral levels were excluded because linear regression analysis could not be performed. Individual swine linear regression of thoracic duct pressure on thoracic vertebral level showed pre-embolization slopes ranging from −0.01 to +0.38 mmHg per vertebral level (median, +0.08 mmHg per vertebral level) (Fig 6, *A*), with the median slope not differing significantly from zero (*P* = .25). Postembolization slopes ranged from −2.21 to +0.08 mmHg per vertebral level (median, −0.20 mmHg per vertebral level) (Fig 6, *B*), and the median slope likewise did not differ significantly from zero (*P* = .09). Overall, a consistent longitudinal cranio-caudal pressure gradient could not be demonstrated.

## Discussion

In this study, paired within-swine comparisons indicated that thoracic duct pressure increased after thoracic duct embolization compared with pre-embolization. The thoracic duct transports approximately 1 to 4 L of lymph unidirectionally per day through valve function and intrinsic smooth-muscle contractions and drains it into the venous system via the venous angle.^2^^,^^16^, ^17^, ^18^ When downstream outflow is restricted, the thoracic duct continues to actively propel lymph from upstream, resulting in excessive inflow and volume loading, which predisposes to an increase in upstream thoracic duct pressure.^19^

When the leakage site is located upstream of the obstruction, the resulting increase in thoracic duct and intralymphatic pressure may exceed the surrounding intrathoracic pressure. This pressure imbalance provides a potential driving force for continued outward lymphatic flow through the disrupted channel and may impede spontaneous closure of the leakage site. In the present study, intrathoracic pressure was not directly measured; therefore, this relationship should be interpreted as a physiological inference rather than a directly demonstrated finding. Consistent with this concept, the elevated upstream intralymphatic pressure associated with thoracic duct obstruction may contribute to the occurrence and persistence of lymphatic leakage both within and outside the operative field.^12^^,^^20^ During esophagectomy for esophageal cancer, the thoracic duct is sometimes resected or ligated en bloc as part of the mediastinal dissection.^21^^,^^22^ Although a direct causal relationship has not been established, thoracic duct ligation has occasionally been followed by refractory chylothorax after surgery.^23^, ^24^, ^25^ Lymphatic leakage has also been reported in pleural spaces remote from the operative field after thoracic duct ligation.^26^ Pressure overload can open and recruit collateral pathways between the thoracic duct or other lymphatic vessels and the venous system, thereby contributing to decompression; however, such collaterals do not always provide sufficient decompression.^12^^,^^24^^,^^27^ When decompression remains inadequate, the collaterals themselves may rupture and become sources of lymphatic leakage.^12^^,^^20^ In addition, pressure overload has been proposed to cause exudative extravasation without overt rupture.^26^^,^^28^^,^^29^ The postembolization rise in thoracic duct pressure observed in this study directly supports the clinical rationale for decompressive interventions such as thoracic duct plasty and thoracic duct–venous bypass, which aim to restore physiological pressure balance and prevent persistent lymphatic leakage. These findings provide a physiological basis for considering decompressive strategies in selected cases of refractory lymphatic leakage following thoracic duct obstruction or embolization. From a physiological perspective, these findings suggest that, in some clinical situations, an occlusive strategy alone may not sufficiently address lymphatic hypertension. Thoracic duct embolization is a well-established and effective treatment option for lymphatic leakage when the leakage site is clearly identified and adequately occluded. Thoracic duct ligation remains the standard treatment for refractory chylothorax, particularly in cases requiring surgical management.^30^ When the site of leakage is clearly identified, ligation effectively eliminates lymphatic flow to the leak point and is highly effective. However, in certain situations, including cases with persistent outflow obstruction or diffuse lymphatic leakage, elevated intralymphatic pressure may remain despite occlusive treatment. In such settings, decompressive strategies such as thoracic duct plasty or thoracic duct–venous bypass may serve as complementary approaches by restoring lymphatic drainage and reducing intralymphatic pressure. In such cases, if embolization does not sufficiently interrupt lymphatic inflow upstream of the leakage site, the resulting thoracic duct outflow obstruction created by the procedure may increase intralymphatic pressure at the leakage site, potentially perpetuating or worsening lymphatic leakage. In such situations, therapeutic strategies that restore or create an effective venous outflow from the thoracic duct may be physiologically advantageous by reducing intralymphatic pressure, including thoracic duct plasty or thoracic duct–venous bypass.

Thoracic duct obstruction can arise not only after surgery or trauma but also from congenital or idiopathic causes.^31^^,^^32^ When thoracic duct obstruction from these various causes persists over the long term, it leads to a sustained increase in upstream lymphatic pressure. This sustained pressure elevation induces dilation of the thoracic duct and lymphatic vessels, valvular insufficiency,^33^ and reflux due to weakening of the normal downstream pressure gradient.^18^ The resulting reflux further increases upstream lymphatic pressure, creating a vicious cycle that promotes additional structural alterations and progressively higher pressures. These pressure elevations may promote or worsen lymphatic leakage without causing overt rupture.^26^^,^^28^^,^^29^

Elevated lymphatic pressure has been suggested to contribute not only to lymphatic leakage but also to lymphedema and abdominal symptoms.^14^^,^^34^, ^35^, ^36^ Decompressive interventions are therefore considered for symptomatic elevations in thoracic duct pressure. Kariya et al reported rapid improvement in chylothorax and chylous ascites caused by thoracic duct obstruction following thoracic duct reconstruction to reopen the obstructed duct.^34^ Other reports indicate that thoracic duct reconstruction and thoracic duct–venous bypass procedures are effective for chylous ascites and chyluria, and have also demonstrated improvement in abdominal symptoms caused by elevated thoracic duct or lymphatic pressure.^9^^,^^13^^,^^15^^,^^37^ In this model, a postembolization pressure gradient in which thoracic duct pressure exceeded central venous pressure was suggested; this finding supports the physiological rationale for decompressive interventions such as thoracic duct plasty and thoracic duct–venous bypass.

Importantly, the finding that thoracic duct pressure exceeded central venous pressure should not be interpreted as a direct mechanism promoting lymphatic leakage. Under normal physiological conditions, a higher thoracic duct pressure relative to central venous pressure facilitates lymphatic drainage into the venous system. Rather, in the present model, this pressure relationship reflects a state of lymphatic hypertension caused by outflow obstruction. In the presence of lymphatic disruption, such elevated intralymphatic pressure may contribute to persistent leakage by maintaining outward flow through injured lymphatic channels.

At the same time, this pressure gradient has important therapeutic implications. Because thoracic duct pressure exceeds central venous pressure, decompressive procedures such as thoracic duct–venous bypass can effectively redirect lymphatic flow into the venous system. Thus, the observed pressure relationship provides a physiological basis for decompressive strategies aimed at restoring lymphatic outflow and reducing intralymphatic pressure.

In this study, no clear longitudinal cranio-caudal pressure gradient along the thoracic duct was detected. Historically, the thoracic duct has been understood to exhibit a cranio-caudal pressure gradient, with higher pressure at the downstream cervical terminus than in more proximal segments.^38^, ^39^, ^40^, ^41^ By contrast, classic physiological studies have reported minimal pressure differences along the thoracic duct.^42^ Although exploratory, our findings are generally consistent with the latter and suggest that a uniformly higher distal pressure should not be assumed.

## Limitations

This study has several limitations. First, it was conducted in an acute swine model under general anesthesia, and the effects of anesthetic agents and mechanical ventilation on the measured thoracic duct and central venous pressures cannot be completely excluded. Second, the sample size was small; in particular, analysis of the longitudinal pressure gradient was limited to four animals with pressure measurements at a restricted number of vertebral levels, which reduced statistical power. Third, the presence of the microcatheter within the thoracic duct may itself have influenced pressure measurements. Fourth, pressures were recorded as visually stable single timepoint values rather than as averages synchronized to respiratory or cardiac cycles, so short-term fluctuations may not have been fully captured. Finally, this study evaluated pressure changes only in the acute phase and did not assess chronic adaptive changes such as collateral pathway formation or pressure redistribution. Because these results were obtained in an acute experimental model, the applicability of these findings to chronic human disease remains uncertain.

## Conclusions

In conclusion, thoracic duct embolization created a pressure gradient in which thoracic duct pressure exceeded central venous pressure in a swine model. This finding provides physiological support for decompressive interventions by establishing a pressure gradient favorable for lymphatic drainage into the venous system, including thoracic duct plasty and thoracic duct–venous bypass as therapeutic strategies for lymphatic leakage associated with thoracic duct outflow obstruction.

## Author Contributions

Conception and design: TM, SK, NT

Analysis and interpretation: TM, MN, YO, YT, KS, NT

Data collection: TM, SK, YU

Writing the article: TM

Critical revision of the article: TM, SK, MN, YU, YO, YT, KS, NT

Final approval of the article: TM, SK, MN, YU, YO, YT, KS, NT

Statistical analysis: TM, MN

Obtained funding: TM, SK, NT

Overall responsibility: SK

## Funding

This work was supported by the Japan Society for the Promotion of Science (JSPS) 10.13039/501100001691KAKENHI—Grant-in-Aid for Scientific Research (C) (Grant Number 22K07680) and Grant-in-Aid for Young Scientists (B) (Grant Number 17K16492). The funding organizations had no involvement in the study design; data acquisition, analysis, or interpretation; manuscript preparation; or the decision to submit for publication.

## Disclosures

None.

## Appendix (online only)

Supplementary docx 1
