# Supplementary material for: Thoracic duct outflow obstruction increases thoracic duct pressure above central venous pressure supporting thoracic duct decompression
Source: JVS Vasc Sci. 2026 May 8;7:100425. doi: 10.1016/j.jvssci.2026.100425 (PMC13254574; doi:10.1016/j.jvssci.2026.100425)
Supplement: Supplementary docx 1 [file mmc1.docx]

Supplementary Table 1. Individual thoracic duct and central venous pressure measurements.

| **Swine number** | **Vessel** | **Phase** | **Thoracic vertebral number** | **Pressure(mmHg)** |
| --- | --- | --- | --- | --- |
| 1 | Thoracic duct | Pre | 12 | 6 |
| 1 | Thoracic duct | Post | 12 | 11 |
| 1 | Central vein | Pre | 12 | 7 |
| 1 | Central vein | Post | 12 | 6 |
| 3 | Thoracic duct | Pre | 12 | 6 |
| 3 | Thoracic duct | Post | 12 | 14 |
| 3 | Central vein | Pre | 12 | 2 |
| 3 | Central vein | Post | 12 | 2 |
| 4 | Thoracic duct | Pre | 12 | 12 |
| 4 | Thoracic duct | Post | 12 | 34 |
| 4 | Central vein | Pre | 12 | 5 |
| 4 | Central vein | Post | 12 | 5 |
| 6 | Thoracic duct | Pre | 5 | 6 |
| 6 | Thoracic duct | Pre | 6 | 7 |
| 6 | Thoracic duct | Pre | 7 | 5 |
| 6 | Thoracic duct | Pre | 9 | 6 |
| 6 | Thoracic duct | Pre | 10 | 6 |
| 6 | Thoracic duct | Pre | 11 | 6 |
| 6 | Thoracic duct | Pre | 12 | 8 |
| 6 | Thoracic duct | Pre | 13 | 8 |
| 6 | Thoracic duct | Post | 5 | 30 |
| 6 | Thoracic duct | Post | 6 | 25 |
| 6 | Thoracic duct | Post | 7 | 23 |
| 6 | Thoracic duct | Post | 9 | 14 |
| 6 | Thoracic duct | Post | 10 | 14 |
| 6 | Thoracic duct | Post | 11 | 14 |
| 6 | Thoracic duct | Post | 12 | 13 |
| 6 | Thoracic duct | Post | 13 | 12 |
| 6 | Central vein | Pre | 5 | 6 |
| 6 | Central vein | Pre | 6 | 6 |
| 6 | Central vein | Pre | 7 | 6 |
| 6 | Central vein | Pre | 8 | 5 |
| 6 | Central vein | Pre | 9 | 4 |
| 6 | Central vein | Pre | 10 | 4 |
| 6 | Central vein | Pre | 11 | 4 |
| 6 | Central vein | Pre | 12 | 4 |
| 6 | Central vein | Post | 5 | 6 |
| 6 | Central vein | Post | 6 | 6 |
| 6 | Central vein | Post | 7 | 6 |
| 6 | Central vein | Post | 8 | 4 |
| 6 | Central vein | Post | 9 | 4 |
| 6 | Central vein | Post | 10 | 4 |
| 6 | Central vein | Post | 11 | 4 |
| 6 | Central vein | Post | 12 | 3 |
| 7 | Thoracic duct | Pre | 6 | 7 |
| 7 | Thoracic duct | Pre | 7 | 7 |
| 7 | Thoracic duct | Pre | 8 | 8 |
| 7 | Thoracic duct | Pre | 9 | 8 |
| 7 | Thoracic duct | Pre | 10 | 8 |
| 7 | Thoracic duct | Pre | 11 | 9 |
| 7 | Thoracic duct | Pre | 12 | 10 |
| 7 | Thoracic duct | Pre | 13 | 9 |
| 7 | Thoracic duct | Post | 6 | 16 |
| 7 | Thoracic duct | Post | 7 | 15 |
| 7 | Thoracic duct | Post | 8 | 15 |
| 7 | Thoracic duct | Post | 9 | 15 |
| 7 | Thoracic duct | Post | 10 | 15 |
| 7 | Thoracic duct | Post | 11 | 15 |
| 7 | Thoracic duct | Post | 12 | 10 |
| 7 | Thoracic duct | Post | 13 | 10 |
| 7 | Central vein | Pre | 5 | 5 |
| 7 | Central vein | Pre | 6 | 5 |
| 7 | Central vein | Pre | 7 | 4 |
| 7 | Central vein | Pre | 8 | 4 |
| 7 | Central vein | Pre | 9 | 3 |
| 7 | Central vein | Pre | 10 | 3 |
| 7 | Central vein | Pre | 11 | 3 |
| 7 | Central vein | Pre | 12 | 3 |
| 7 | Central vein | Pre | 13 | 3 |
| 7 | Central vein | Post | 5 | 7 |
| 7 | Central vein | Post | 6 | 6 |
| 7 | Central vein | Post | 7 | 5 |
| 7 | Central vein | Post | 8 | 7 |
| 7 | Central vein | Post | 9 | 3 |
| 7 | Central vein | Post | 10 | 3 |
| 7 | Central vein | Post | 11 | 4 |
| 7 | Central vein | Post | 12 | 4 |
| 7 | Central vein | Post | 13 | 4 |
| 8 | Thoracic duct | Pre | 5 | 6 |
| 8 | Thoracic duct | Pre | 6 | 5 |
| 8 | Thoracic duct | Pre | 7 | 6 |
| 8 | Thoracic duct | Pre | 8 | 5 |
| 8 | Thoracic duct | Pre | 9 | 6 |
| 8 | Thoracic duct | Pre | 10 | 6 |
| 8 | Thoracic duct | Pre | 11 | 6 |
| 8 | Thoracic duct | Pre | 12 | 6 |
| 8 | Thoracic duct | Pre | 13 | 6 |
| 8 | Thoracic duct | Post | 5 | 10 |
| 8 | Thoracic duct | Post | 6 | 11 |
| 8 | Thoracic duct | Post | 7 | 10 |
| 8 | Thoracic duct | Post | 8 | 10 |
| 8 | Thoracic duct | Post | 9 | 10 |
| 8 | Thoracic duct | Post | 10 | 10 |
| 8 | Thoracic duct | Post | 11 | 10 |
| 8 | Thoracic duct | Post | 12 | 10 |
| 8 | Thoracic duct | Post | 13 | 11 |
| 8 | Central vein | Pre | 5 | 5 |
| 8 | Central vein | Pre | 6 | 5 |
| 8 | Central vein | Pre | 7 | 5 |
| 8 | Central vein | Pre | 8 | 4 |
| 8 | Central vein | Pre | 9 | 4 |
| 8 | Central vein | Pre | 10 | 5 |
| 8 | Central vein | Pre | 11 | 5 |
| 8 | Central vein | Pre | 12 | 4 |
| 8 | Central vein | Pre | 13 | 3 |
| 8 | Central vein | Post | 5 | 4 |
| 8 | Central vein | Post | 6 | 4 |
| 8 | Central vein | Post | 7 | 4 |
| 8 | Central vein | Post | 8 | 4 |
| 8 | Central vein | Post | 9 | 3 |
| 8 | Central vein | Post | 10 | 3 |
| 8 | Central vein | Post | 11 | 5 |
| 8 | Central vein | Post | 12 | 3 |
| 8 | Central vein | Post | 13 | 3 |
| 9 | Thoracic duct | Pre | 9 | 7 |
| 9 | Thoracic duct | Pre | 10 | 6 |
| 9 | Thoracic duct | Post | 9 | 13 |
| 9 | Thoracic duct | Post | 10 | 10 |
| 9 | Central vein | Pre | 9 | 11 |
| 9 | Central vein | Pre | 10 | 9 |
| 9 | Central vein | Post | 9 | 9 |
| 9 | Central vein | Post | 10 | 7 |
| 10 | Thoracic duct | Pre | 5 | 8 |
| 10 | Thoracic duct | Pre | 6 | 9 |
| 10 | Thoracic duct | Pre | 7 | 9 |
| 10 | Thoracic duct | Pre | 8 | 8 |
| 10 | Thoracic duct | Pre | 9 | 8 |
| 10 | Thoracic duct | Pre | 10 | 8 |
| 10 | Thoracic duct | Pre | 11 | 8 |
| 10 | Thoracic duct | Pre | 12 | 9 |
| 10 | Thoracic duct | Post | 5 | 13 |
| 10 | Thoracic duct | Post | 6 | 13 |
| 10 | Thoracic duct | Post | 7 | 13 |
| 10 | Thoracic duct | Post | 8 | 11 |
| 10 | Thoracic duct | Post | 9 | 12 |
| 10 | Thoracic duct | Post | 10 | 11 |
| 10 | Thoracic duct | Post | 11 | 12 |
| 10 | Thoracic duct | Post | 12 | 12 |
| 10 | Central vein | Pre | 6 | 3 |
| 10 | Central vein | Pre | 7 | 5 |
| 10 | Central vein | Pre | 8 | 5 |
| 10 | Central vein | Pre | 9 | 4 |
| 10 | Central vein | Pre | 10 | 4 |
| 10 | Central vein | Pre | 11 | 4 |
| 10 | Central vein | Pre | 12 | 3 |
| 10 | Central vein | Pre | 13 | 3 |
| 10 | Central vein | Post | 6 | 7 |
| 10 | Central vein | Post | 7 | 7 |
| 10 | Central vein | Post | 8 | 7 |
| 10 | Central vein | Post | 9 | 6 |
| 10 | Central vein | Post | 10 | 5 |
| 10 | Central vein | Post | 11 | 5 |
| 10 | Central vein | Post | 12 | 4 |
| 10 | Central vein | Post | 13 | 4 |

Foot note. This table provides all individual thoracic duct and central venous pressure measurements obtained in each swine before and after thoracic duct embolization, including measurements at each thoracic vertebral level. These data correspond to the values used for all statistical analyses.
